# 

**Published:** 1893-08

**Authors:** 


### CONTENTS FOR AUGUST, 1893.

PAGE.

ORIGINAL ADDRESS.

The Prevention of Disease. By DeLancey Rochester, M. D. .... 1

ORIGINAL COMMUNICATIONS.

Puerperal Sepsis; Its Prevention and Cure. By William Warren Potter, M. D. .... 10

CLINICAL LECTURE.

Exploratory Laparatomy—Pregnant Uterus. By Matthew D. Mann, M. D. .... 19

SOCIETY PROCEEDINGS.

Medical Society of the County of Erie .... 22
Philadelphia County Medical Society—Disputed Points in Hysterectomy .... 26

TRANSLATION.

Lysol .... 37

SELECTION.

The Spelling of Some Medical Words .... 42

EDITORIAL.

Spelling of Medical Words .... 47

Topics of the Month .... 47

Personal .... 48
Obituary .... 50
Academy of Medicine Notes .... 52
Society Meetings .... 53
Book Reviews .... 54
Literary Notes .... 61
Miscellany .... 62

### Index to Advertisements.

PAGE.

PAGE.

